# Supplementary material for: A pragmatic randomized controlled trial reports lack of efficacy of hydroxychloroquine on coronavirus disease 2019 viral kinetics
Source: Nat Commun. 2020 Oct 20;11:5284. doi: 10.1038/s41467-020-19056-6 (PMC7576792; doi:10.1038/s41467-020-19056-6)
Supplement: Supplementary file 1 — Supplementary Information [file 41467_2020_19056_MOESM1_ESM.pdf]

SUPPLEMENTARY INFORMATION

A pragmatic randomized controlled trial reports lack of efficacy of hydroxychloroquine on coronavirus disease 2019 viral kinetics

Magnus Nakrem Lyngbakken[1,2], Jan-Erik Berdal[2,3], Arne Eskesen[3], Dag Kvale[2,4], Inge Christoffer Olsen[5], Corina Silvia Rueegg[5,6], Anbjørg Rangberg[7], Christine Monceyron Jonassen[7], Torbjørn Omland[1,2], Helge Røsjø[2,8], Olav Dalgard[2,3]

[1]Division of Medicine, Akershus University Hospital, Lørenskog, Norway

[2]Institute of Clinical Medicine, Faculty of Medicine, University of Oslo, Oslo, Norway

[3]Department of Infectious Diseases, Division of Medicine, Akershus University Hospital, Lørenskog, Norway

[4]Department of Infectious Diseases, Oslo University Hospital, Oslo, Norway

[5]Department of Research Support for Clinical Trials, Oslo University Hospital, Oslo, Norway

[6]Oslo Centre for Biostatistics and Epidemiology, Oslo University Hospital, Oslo, Norway

[7]Center for Laboratory Medicine, Østfold Hospital Trust, Grålum, Norway

[8]Division of Research and Innovation, Akershus University Hospital, Lørenskog, Norway

Contents

(1) Supplementary Methods

(2) Supplementary Tables

(3) Supplementary Figures

Supplementary Methods

Procedures for sampling and SARS-CoV-2 viral loads quantitation

Oropharyngeal swab samples were taken from patients at inclusion, at 48 hours and at 96 hours, by a selected group of study physicians. Nasopharyngeal sampling was planned in the initial study protocol, but due to trial conduction in the middle of a global pandemic and an immediate shortage of nasopharyngeal swabs for anything but clinical Covid-19 diagnostics, the study steering committee decided on oropharyngeal sampling using chlamydia swabs. The swabs were rotated for ten seconds on the posterior oropharyngeal mucosal membrane (over both tonsils, soft palate and posterior oropharynx), placed in 1 mL universal in-house virus transport medium, and kept frozen at -80°C until analysis. We validated the oropharyngeal versus nasopharyngeal sampling in seven SARS-CoV-2 positive patients and three SARS-CoV-2 negative healthy controls. There was total agreement in SARS-CoV-2 qualitative results from paired samples taken either from nasopharynx as for routine diagnosis or from oropharynx with sampling devices used for this study. The mean pairwise difference in Ct values in the SARS-CoV-2 positive samples was of 2.5, with stronger PCR positive results observed in the samples collected with the devices used for this study (**Supplementary Table 4**). The universal in-house virus transport medium was prepared as follows: Two grams of bovine serum albumin (IgG free, protease free) was added to 50mL of phosphate-buffered saline, sterile filtered, and added to sterile distilled water (to 400mL). 200mL of sterile phenol red solution was then added for a total volume of 600mL. pH was adjusted with 1 molar sodium hydroxide for a final pH of 7.48 to 7.50.

Supplementary Table 1. Clinical status on day 14 after randomization (n=51).

	Dead	Hospitalized, on invasive mechanical ventilation or extracorporeal membrane oxygenation	Hospitalized, on non-invasive ventilation or high flow oxygen devices	Hospitalized, requiring supplemental oxygen	Hospitalized, not requiring supplemental oxygen	Not hospitalized, but unable to resume normal activities	Not hospitalized, with resumption of normal activities	Odds ratio (95% confidence interval)*
HCQ + SOC (n=26)	1 (3.8%)	0	1 (3.8%)	0	1 (3.8%)	3 (11.5%)	20 (76.9%)	1.11 (0.31 to 4.01)
SOC (n=25)	1 (4.0%)	1 (4.0%)	0	2 (8.0%)	0	2 (8.0%)	19 (76.0%)	

HCQ, hydroxychloroquine. SOC, standard care. * Cumulative logistic regression.

Supplementary Table 2. Number of available NEWS assessments per day

Time point	Standard care (n=25)	Hydroxychloroquine therapy plus standard care (n=26)	Total (n=51)
Randomisation	25	26	51
24 hours	25	26	51
48 hours	15	22	37
72 hours	11	16	27
96 hours	8	10	18

Supplementary Table 3. Sample size calculations for increasing effect size and constant standard deviations (with power of 0.8 and 0.9) based on results from the NO COVID-19 Study.

Alpha	Power	N	N1	N2	Delta	M1	M2	SD1	SD2
0.05	0.80	1080	540	540	-0.1	-0.1	-0.2	0.6055	0.565
0.05	0.80	272	136	136	-0.2	-0.1	-0.3	0.6055	0.565
0.05	0.80	122	61	61	-0.3	-0.1	-0.4	0.6055	0.565
0.05	0.80	70	35	35	-0.4	-0.1	-0.5	0.6055	0.565
0.05	0.80	46	23	23	-0.5	-0.1	-0.6	0.6055	0.565
0.05	0.80	32	16	16	-0.6	-0.1	-0.7	0.6055	0.565
0.05	0.80	26	13	13	-0.7	-0.1	-0.8	0.6055	0.565
0.05	0.80	20	10	10	-0.8	-0.1	-0.9	0.6055	0.565
0.05	0.80	16	8	8	-0.9	-0.1	-1.0	0.6055	0.565
0.05	0.90	1444	722	722	-0.1	-0.1	-0.2	0.6055	0.565
0.05	0.90	364	182	182	-0.2	-0.1	-0.3	0.6055	0.565
0.05	0.90	164	82	82	-0.3	-0.1	-0.4	0.6055	0.565
0.05	0.90	94	47	47	-0.4	-0.1	-0.5	0.6055	0.565
0.05	0.90	60	30	30	-0.5	-0.1	-0.6	0.6055	0.565
0.05	0.90	44	22	22	-0.6	-0.1	-0.7	0.6055	0.565
0.05	0.90	32	16	16	-0.7	-0.1	-0.8	0.6055	0.565
0.05	0.90	26	13	13	-0.8	-0.1	-0.9	0.6055	0.565
0.05	0.90	20	10	10	-0.9	-0.1	-1.0	0.6055	0.565

Alpha, significance level set constant to 0.05; N, total study sample; N1, sample in group 1; N2, sample in group 2; Delta, difference in the viral load slope between intervention and control group; M1, viral load slope of the control group (set constant to -0.1 based on the effect in the NO COVID-19 Study); M2, viral load slope of the intervention group (varying from -0.2 to -1.0); SD1, standard deviation of M1 (set constant to 0.6055 based on the SD in the NO COVID-19 Study); SD2, standard deviation of M2 (set constant to 0.5650 based on the SD in the NO COVID-19 Study).

Supplementary Table 4. Validation of oropharyngeal versus nasopharyngeal virus sampling for SARS-CoV-2.

		Ct values	
	ID	A	B
SARS-CoV-2 positive patients (1-7)	1	44.67	36.88
	2	Negative	Negative
	3	30.77	28.61
	4	35.28	35.73
	5	Negative	Negative
	6	36.93	35.78
	7	25.05	23.13
SARS-CoV-2 negative healthy controls (1-3)	1	Negative	Negative
	2	Negative	Negative
	3	Negative	Negative

A, routine nasopharyngeal sampling. B, oropharyngeal sampling with chlamydia swab in universal in-house virus transport medium.

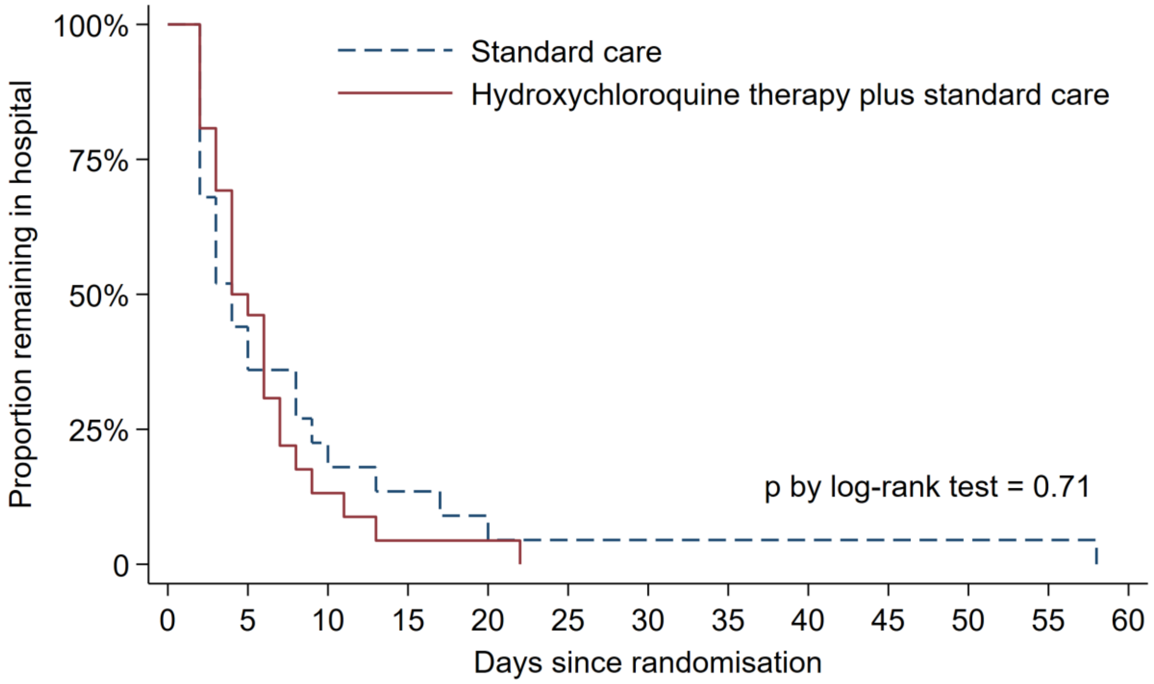

Supplementary Figure 1. Kaplan-Meier plot for duration of hospital admission (n=51).

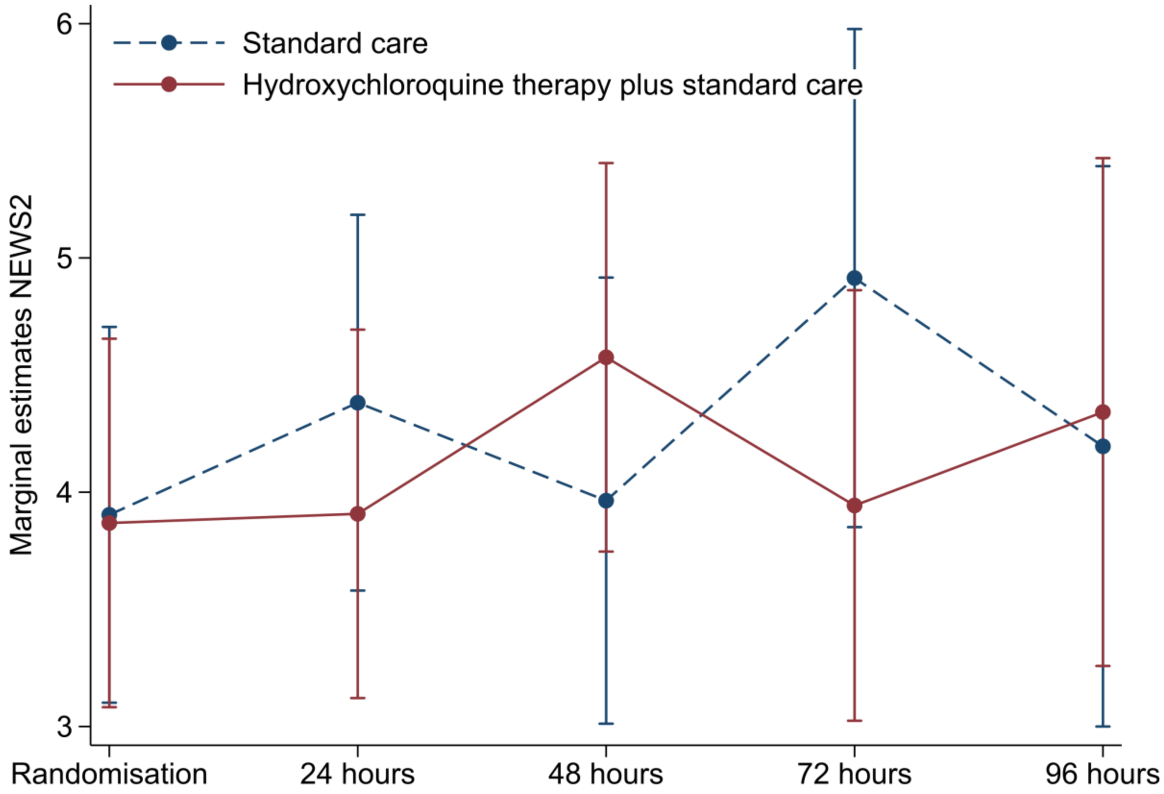

Supplementary Figure 2. Change in estimated mean NEWS2 from randomisation to 96 hours. Plot displays estimated marginal means (dots) and 95% confidence intervals (error bars). NEWS2, National Early Warning Score 2.

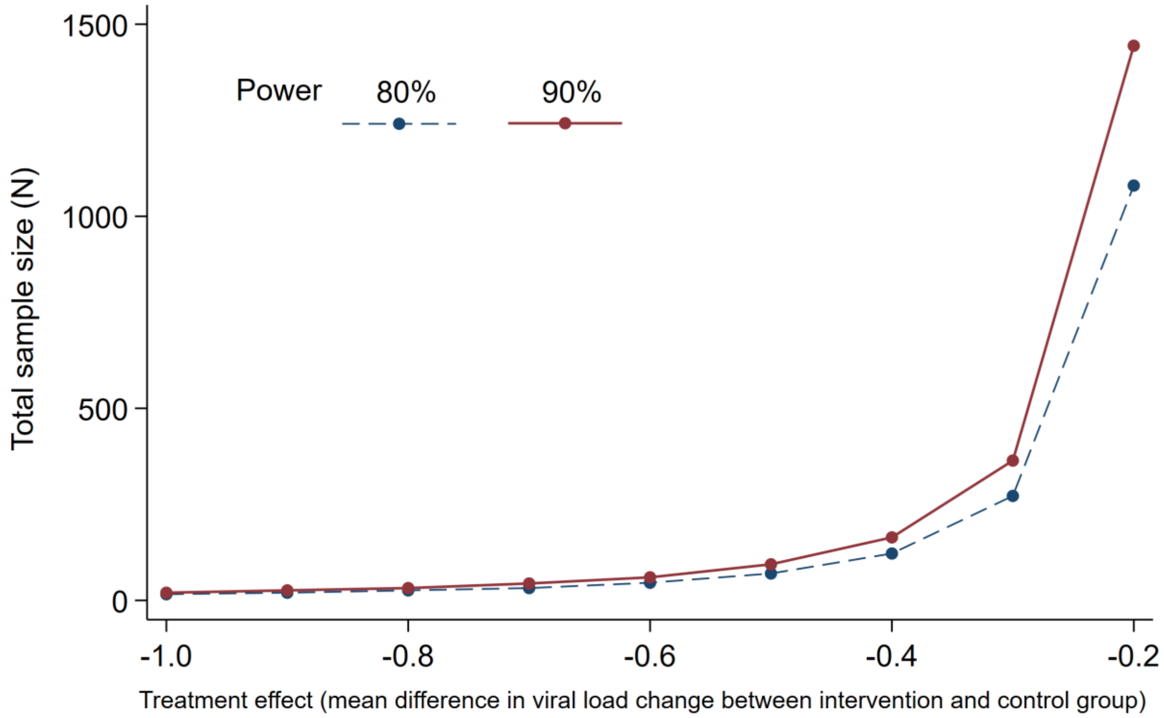

Supplementary Figure 3. Estimated total sample size for a two-sample means test.
Constant parameters: $\alpha = 0.05$, $\mu_1 = -0.1$, $\sigma_1 = 0.61$, $\sigma_2 = 0.56$.
